# Psychological Distress and Problem Gambling in Elite Athletes during COVID-19 Restrictions—A Web Survey in Top Leagues of Three Sports during the Pandemic

**DOI:** 10.3390/ijerph17186693

**Published:** 2020-09-14

**Authors:** Anders Håkansson, Caroline Jönsson, Göran Kenttä

**Affiliations:** 1 Faculty of Medicine, Depterment of Clinical Sciences Lund, Psychiatry, Lund University, S-22100 Lund, Sweden; 2 Malmö Addiction Center, Clinical Sports and Mental Health Unit, S-20502 Malmö, Sweden; caroline.jonsson@skane.se; 3 FIFPRO (global representative for professional football players), Scorpious 161, NL-2132LR Hoofdorp, The Netherlands; 4 Spelarföreningen, Sweden (national representative for football players), Göteborgsvägen 84B, S-433 63 Sävedalen, Sweden; 5 The Swedish School of Sport and Health Sciences, S-114 86 Stockholm, Sweden; goran.kentta@gih.se; 6 School of Human Kinetics, University of Ottawa, Ottawa, ON K1N 6N5, Canada; 7 Swedish Sport Federation, S-100 61 Stockholm, Sweden

**Keywords:** COVID-19, elite athlete, depression, anxiety, sport, gambling disorder, problem gambling, pandemic, crisis

## Abstract

COVID-19 and lockdown strategies may affect mental health and addictive behavior differently in the population, and elite athletes are among the professions clearly affected by the pandemic. This study in top elite athletes aimed to study current perceived psychological influence from COVID-19 and symptoms of depression, anxiety and changes in alcohol drinking, gambling behavior and problem gambling in the midst of the COVID-19 lockdown. This web survey included athletes in top leagues of soccer, ice hockey and handball in Sweden (N = 327, 62% men). A total of 66% and 51% were worried about the future of their sport or about their own future in sports, respectively. Feeling worse psychologically during the pandemic was common (72% of women, 40% of men, *p* < 0.001); depression criteria were endorsed by 19% of women and three percent of men (*p* < 0.001); anxiety criteria by 20% of women and five percent of men (*p* < 0.001). Reporting increased gambling during the pandemic was associated with gambling problem severity. Moderate-risk or problem gambling was seen in 10% of men and none of the women (*p* < 0.001). Depression and anxiety were associated with feeling worse during the COVID-19 pandemic and with concern over one’s own sports future. In conclusion, COVID-19-related distress is common in elite athletes and associated with mental health symptoms. Gambling increase during the pandemic was rare, but related to gambling problems, which were common in male athletes. The calls for increased focus on COVID-19-related concerns in athletes and on problem gambling in male athletes.

## 1. Introduction

Researchers have expressed concerns regarding mental health consequences of the COVID-19 pandemic, related to different kinds of mental health in the general population [1,2,3,4]. In addition to the overall impact on society, some sectors and professional settings in society are likely to be more affected than others by the pandemic and by the confinement and other restrictions applied in order to limit the spread of the infection. Such impact may be caused by feelings of worry related to one’s employment situation and such mental health consequences may be very diverse across different professions. Recently, findings have demonstrated that insecurity about one’s job situation and financial concerns due to COVID-19 can cause mental health symptoms [5].

One of the professional groups most clearly affected by the COVID-19 pandemic are elite athletes, due to the dramatic changes to the world of sports during the pandemic. Due to COVID-19-related restrictions, and in many cases actual confinement, competitions have been largely cancelled or postponed with most uncertain plans for the future, including the interruption of ongoing seasons or the cancellation or postponement of major events such as the male European EURO 2020 soccer championship and the female EURO 2021 soccer championship, female and male ice hockey world championships and the 2020 Tokyo summer Olympics, including two of the sports included in the present study. This vast impact on sports calls for action within the world of sports and requires research in order to understand the true impact of these changes [6], beyond the need to prevent physical consequences in elite athletes [7]. It has been previously established that elite athletes may face specific risks of poor mental health, either due to characteristics of the world of sports including severe demands or because of maintenance or worsening of pre-existing symptoms [8,9]. Although the prevalence rates of mental health disorders have not demonstrated to be higher, an elite level athlete who shows signs of poor mental health is still associated with stigma and at risk of misperceptions of mental health symptoms by health care practitioners since athletes typically are in seemingly good physical condition in contrast to a typical patient [9]. While definitions of the elite athlete group differ in the literature, elite athletes are typically described as athletes competing on a national or international level, including athletes competing in national leagues [10]. Due to the profound impact of COVID-19 on sports, the player unions of elite players in major team sports in Sweden took an initiative, in collaboration with the present research group, to assess COVID-19-related consequences on mental health in their members in May, 2020, during the pandemic and during total sports lockdown.

Potential consequences of the COVID-19-related lockdown in elite athletes may include both sub-clinical symptoms of worry and uncertainty and more clinically significant symptoms. Recently, media reports of a European survey in elite soccer players revealed markedly increased rates of depression and anxiety in soccer players during the COVID-19 pandemic, compared to a recent survey carried out prior to this crisis [11]. Likewise, addictive behavior is one of the mental health issues raised by researchers with respect to the COVID-19 pandemic [12,13,14], including an increased risk of alcohol problems, drug problems and behavioral addictions including problem gambling [15]. For example, researchers have expressed the concerns that alcohol consumption may be increased by the pandemic [16] and early data demonstrate that a significant minority of the population may have increased their consumption in line with other changes in everyday lives [17]. While substance use disorders are typically likely to be less prevalent in adult elite athletes during their active career [9], concerns have been raised about a likely increased risk in problem gambling. Problem gambling is known to be higher in elite athletes, at least in male athletes, than in the general population [18,19,20], making problem gambling particularly important to assess in that population.

Problem gambling is a well-established addictive condition, with a prevalence ranging from well below one percent to almost six percent in different settings world-wide [21]. Risk factors of problem gambling reported include male gender, younger age, lower level of education, other mental health problems, addictive behaviors and contextual risk factors such as disadvantaged living conditions [22,23]. Moreover, problem gambling is known to be enhanced by psychological risk factors such as cognitive erroneous belief including a loss of control despite an illusion of control derived from the technical design of many games [22,24]. A well-established model for the development of a disordered gambling pattern is the pathways model, which demonstrates three typical trajectories to problem gambling (behaviorally conditioned, emotionally vulnerable and antisocial/impulsive gamblers), for whom the reasons for gambling and types of gambling patterns have been shown to differ [25,26].

Specific concerns about gambling during the COVID-19 pandemic have been associated with more time spent online when at home [27], increased gambling in the context of increased mental distress and the potential change in gambling patterns when the world of sports closed down, hypothetically favoring other rapid, high-access online gambling types [15], altogether concerns leading to policy interventions by governments in several countries [28,29,30]. A recent general population study—in the same geographical setting as the one assessed here—demonstrated that a minority of respondents reported an increase in gambling during the pandemic, and although these respondents were fewer than those reporting a decrease, their rates of problem gambling were markedly higher than in others [31].

Altogether, based on the obviously profound impact on the working conditions of elite athletes, and following a national player union initiative, the present study addressed top elite athletes in the highest leagues of soccer, handball and ice hockey in Sweden. Based on the potential impacts on the group described above, the study aimed to assess, during the ongoing crisis and its major consequences on society, how COVID-19 may have affected this group with respect to: (1) measures of depression and anxiety, (2) self-reported worry about one’s career and one’s sport, (3) self-reported changes in alcohol drinking, and (4) self-reported changes in gambling and measures of problem gambling, as well as whether these symptoms are related to one another and to demographic data or type of sports. The authors hypothesized that worry about one’s sports and one’s own future in sports, as well as current psychological distress during the pandemic would be common and that anxiety and depression would correlate with these symptoms of worry and distress. In addition, it was hypothesized that gambling and alcohol consumption may have increased in a substantial proportion of participants, and that all these potential changes would be related to gender and to one another. In addition, it was hypothesized that problem gambling would differ with respect to gender.

## 2. Methods

The present study was an anonymous web survey study addressing elite athletes in the clubs of the two highest women’s and men’s leagues in soccer and ice hockey, and the highest leagues of male and female handball. The survey was sent to the members by each of the players’ unions, with information about the study and an offer to participate, including a web link to the survey, which was opened only if the individual provided informed consent electronically. When the survey was filled out by an individual, her/his responses were sent to the researchers, through the companies Patient Information Broker (PIB) and I-Mind Consulting, which carried out the technical structure of the questionnaire and the actual data collection. The researchers, as well as the companies providing the technical data collection, were entirely blinded to the identity of the respondents.

The project was reviewed by the Swedish Ethical Review Authority (file number 2020–02,290), which concluded that the project did not require formal ethical approval as it does not include identified personal data and expressed that its only ethical advice were to explicitly address only athletes from the age of 15 years, and to structure the information to participants according to instructions available from the ethical review authority. For this reason, the survey was sent only to members aged 15 years of above (as ice hockey players typically in the female teams anecdotally are around that age, although a current search on the top league female ice hockey teams found only eight players aged 15 years and none younger than this), and the 15-year age limit was also explicitly stated in the information to potential participants. In that information, it was also explicitly stated that the survey should be filled out only once, and that the invitation was personal.

### 2.1. Participants

The survey was sent by e-mail, directly only to players who were union members; 487 soccer players (70% men), 140 handball players (67% men) and 518 ice hockey players (96% men), in total 1145 players (82% men). Surveys were sent on May 20 to all soccer players, from May 21 to handball players and from May 26 to ice hockey players, and the survey closed on June 10. Two reminders were sent to union members in soccer, whereas one single reminder was sent to the ice hockey players and handball players (due to a perceived relative saturation of response in close association with the first and the second distributions). In order to increase representativity, in addition to the direct e-mail to union members, the survey was also distributed to representatives of each of the clubs in the two top leagues in male soccer and the top league in female soccer (in total 44 clubs) and to representatives of clubs in the top leagues of female ice hockey (in total 10 clubs, Table 1).

### 2.2. Setting

The study was carried out during a period of time with full restrictions on competing sports activities in Sweden throughout the study period, whereas at the end of the data collection, on May 29, it was announced that the top leagues of Swedish soccer were to start on June 15. Throughout the study period, Sweden maintained restrictions enforced since March 2020, with a ban on public activities involving 50 individuals or more [32], virtually closing down all sports activities except low-tier friendly games and child and youth activities [33]. Regarding gambling, this is increasingly taking place online in the present setting, with online casino and online sports betting being the two leading gambling types reported by problem gamblers seeking treatment [34].

### 2.3. Measures

Sociodemographic data: In order to fully anonymize responses, partly because many potential participants can be well-known to the general public, sociodemographic data only included gender and age, the latter reported only as either below 25 years or 25 years or older.

COVID-19-related changes in psychological distress, alcohol and gambling: Questions were asked about changes in psychological health during the COVID-19 pandemic, following a brief description of the consequences the COVID-19 pandemic has on society; ‘since these changes in Sweden started, has your psychological health changed?’ (‘better’, ‘unaffected’, ‘slightly worse’ or ‘much worse’). Following the same vignette, questions were asked about whether the individual feels worried about the future of one’s sport in Sweden (‘not worried’, ‘unsure’, ‘slightly worried’ or ‘very worried’) and about one’s own future in the sport (‘not worried’, ‘unsure’, ‘slightly worried’, ‘very worried’). Again, following the same vignette, with respect to the sports career, a question was asked about whether the respondent had planned for dual careers, i.e., a different career in addition to the active sports career (‘which are your thoughts about a dual career, i.e., studying or working on a different career apart from sports?’: ‘planned for dual careers both before and during the pandemic’, ‘started to plan for dual careers in response to the pandemic’, ‘planned for dual careers before, but more concerned now about the other career’, ‘have never planned for dual careers’ or ‘uncertain/wish not to answer’).

Questions were asked about self-reported changes in alcohol consumption during the pandemic, using the COVID-19 situation for background; ‘since these changes started in Sweden, has you’re alcohol consumption changed?’ (‘drink less’, ‘unaffected’, ‘drink more’ or ‘do not drink, neither now nor prior to the pandemic’). With the same vignette and the corresponding wording, the next question asked about potential changes in gambling habits during the pandemic (‘gamble less’, ‘unaffected’, ‘gamble more’ or ‘do not gamble, neither now nor prior to the pandemic’). Here, the meaning of gambling was specified, mentioning several common gambling types and with the specification that these may be land-based or online-based. In addition, after a sentence stating the changes to the world of sports during the pandemic, a question was asked about how these changes to sports-related gambling specifically had affected gambling patterns (‘gamble more on other sports’, ‘gamble more on online casino’, ‘gamble more on horse race betting’, ‘gamble more on other games’, ‘gamble less overall’ or ‘unaffected, do not gamble on sports betting’). Questions on changes in alcohol and gambling behavior were worded in the same way as in the present COVID-19-related study from the same setting [31].

Symptoms of depression and anxiety: In order to facilitate comparisons, symptoms of depression and anxiety were measured with the same instruments as in a recent survey carried out in soccer players and made available in news media [8]. Depressive symptoms were measured using the PHQ-9 (patient health questionnaire-9, [35]), and anxiety symptoms were measured using the GAD-7 [31]. Both for the PHQ-9 and the GAD-7, the cutoff value of 10 or more was used, corresponding to a level of moderate severity for both depression and anxiety, respectively [35,36]. Cutoff levels were chosen to correspond to a recent study using the same instruments in a corresponding sample of elite athletes in Sweden [37] and to the recent survey in a number of European countries assessing soccer players during the COVID-19 pandemic, hitherto reported in a report in the media [11].

Symptoms of problem gambling: Gambling problems were measured using the problem gambling severity index (PGSI, [38]), a nine-item scale assessing core symptoms of problem gambling with a past-12-month perspective in the questions asked. For the PGSI, the categorization used was the same one as in general population studies in the present setting; a total score of 0 was considered to represent no risk gambling, 1–2, low-risk gambling, 3–7, moderate-risk gambling and 8 or above represented problem gambling. As in previous publications from the present setting, moderate-risk gambling and problem gambling were collapsed into one category [39,40].

### 2.4. Statistical Methods

In univariate analyses, individuals with or without fulfilled depression or anxiety, respectively, were compared to the remaining individuals with respect to age, gender, increased alcohol consumption or gambling and variables associated with COVID-19-related concerns, using chi-squared analyses (or Fisher’s exact test whenever the absolute number of individuals in any one category was below five). For ordinal scale variables, the linear-by-linear chi-squared measure was used. For depression and anxiety, comparisons were repeated as a logistic regression involving each potential correlate controlling for gender, due to the strong gender association with anxiety and depression in the study. The same comparisons were made for the comparison of those increasing their gambling and their alcohol consumption, respectively, compared to other respondents. Here, the level of gambling severity was also compared across the two groups. For all analyses, *p* values below 0.05 were considered significant. In Table 2, numbers of missing data for all variables are displayed. For the outcome measures controlling for gender (increased gambling, increased alcohol consumption, depression, anxiety and moderate-risk/problem gambling), gender was available for all individuals with available data for the study variable. No data were imputed.

## 3. Results

A total of 327 responses were received, among which 191 from soccer players, 84 from ice hockey players and 52 from handball players. Thus, response rates in comparison to the number of directly addressed union members were 39% in soccer players, 16% in ice hockey players and 37% in handball players. A total of 310 individuals reported gender and age; among them, 192 (62%) were men and 118 were women. Men represented 54% of responding soccer players, 87% of ice hockey players and 50% of handball players (Table 1). A total of 179 (58%) were 25 years of older, whereas 131 were younger than 25 years of age. Sample characteristics and survey answers are displayed in Table 2.

### 3.1. COVID-19-Related Mood and Concerns

A total of 46% (n = 135) of respondents (from a total of 296 responses) reported feeling psychologically slightly worse during the pandemic, whereas 6% (n = 18) reported feeling much worse. Those feeling at least slightly worse represented 60% of soccer players, 33% of ice hockey players and 55% of handball players. Among 295 respondents, six (n = 17) and 60% (n = 178) reported being very worried or slightly worried about the future of their sport, respectively. Those at least slightly worried represented 64% of soccer players, 73% of ice hockey players and 63% of handball players. Nine percent (n = 27) and 42% (n = 123), respectively, were very worried or slightly worried about their own future in their sport, respectively. Those reporting at least being slightly worried about their own situation in sports represented 56% of soccer players, 40% of ice hockey players and 50% of handball players. Among 295 respondents, 48% (n = 143) reported having planned for a dual career both before and during the COVID-19 pandemic, besides the sports activity, nine percent (n = 27) reported having started to plan for this in response to the COVID-19 pandemic and 11% (n = 33) had planned for a dual career, but currently felt more concerned about the non-sports career, and 11% (n = 33) were uncertain. Twenty percent (n = 59) reported having no plan for a dual career, neither before nor during the COVID-19 crisis (the latter reported by 17% of soccer players, 10% of handball players and 33% of ice hockey players answering this question).

Feeling at least slightly worse during the pandemic was significantly more common in women (72%) than in men (40%, *p* < 0.001); 13% of women (n = 14) reported feeling much worse and 60% (n = 65) slightly worse during the pandemic, whereas among men, 2% (n = 4) reported feeling much worse and 37% (n = 70) reported feeling slightly worse.

Among women, 14% (n = 15) were very worried and 45% (n = 49) slightly worried about their own future in sports, whereas the same figures for men were 6% (n = 12) and 40% (n = 74), respectively. Altogether, being at least slightly worried about one’s own future in sports was significantly more common in women than in men (59% vs. 46%, *p* = 0.03). Among women, 11% (n = 12) reported being very worried and 57% (n = 62) slightly worried about the future of their sport overall, whereas the corresponding figures for men were 3% (n = 5) and 62% (n = 116), respectively. Altogether, feeling worried about the future of one’s sport overall (69% of women and 65% of men) was unrelated to gender (*p* = 0.51).

### 3.2. Anxiety and Depression during the COVID-19 Pandemic

Among those with available responses (n = 286), anxiety criteria were met by 10% (n = 30) and for depression (from a total of 279 responses), 9% (n = 25) met the criteria. Both disorders were significantly more common in women than in men (20% vs. 5% anxiety, *p* < 0.001 and 19 vs. 3% for depression, *p* < 0.001).

Respondents who endorsed depression criteria or anxiety criteria, respectively, were significantly more likely to report worse psychological mood during the COVID-19 crisis (<0.001) and significantly more likely to report worry about their own future in the sport (*p* < 0.001), both also significant when controlling for gender, whereas both depression and anxiety were unrelated to age, type of sport, overall worry about the future of their sport or increased alcohol consumption or gambling (Table 3 and Table 4).

### 3.3. Gambling and Alcohol Drinking in Response to the COVID-19 Pandemic

Among 277 individuals, 16% (n = 45) reported drinking more alcohol during the pandemic and 7% (n = 20) reported gambling more. Having increased gambling during COVID-19 was reported by 9% of men (n = 16) and 4% (n = 4) of women (*p* = 0.15, Fisher’s exact test) and endorsing the ‘no, I don’t gamble at all’ option was markedly more common in women (57%, n = 67) than in men (35%, n = 67, *p* < 0.001). For 274 individuals, problem gambling data (PGSI) was available. 86% had no problem risk (n = 236), 7% were low-risk gamblers (n = 20), 5% (n = 15) were moderate-risk gamblers and 1% (n = 3) were problem gamblers. Moderate-risk or problem gambling was endorsed by 10% of men (n = 18) and by zero women (*p* < 0.001, Fisher’s exact test). Among the 274 respondents answering the specific question about how restrictions in sport and sports betting specifically had affected their gambling behavior during the COVID-19 pandemic, 1% (n = 2) reporting gambling more on other types of sports betting than before, 1% (n = 4) reported increased online casino gambling, 7% (n = 19) reported increased horse race betting, 1% (n = 4) reported gambling more on other games, 18% (n = 48) reported gambling less and 73% (n = 201) reported not gambling on sports and therefore unaffected by this change.

Having increased gambling during the crisis was strongly associated with higher gambling severity *(p <* 0.001, significant even after controlling for gender, *p* < 0.01, data not shown), but unrelated to other covariates tested (Table 5). Having increased alcohol consumption during the COVID-19 crisis was significantly associated with worry about the future of one’s sport (*p* = 0.01), whereas this association was no longer seen when controlling for gender (*p* = 0.17) and increased alcohol consumption was unrelated to other covariates tested (Table 6).

## 4. Discussion

The present self-report survey study, in elite athletes in top leagues of three sports in Sweden, aimed to study self-reported distress due to COVID-19 and symptoms of depression, anxiety, gambling problems and changes in gambling and alcohol consumption, in the midst of the pandemic and the restrictions to the world of sports associated with it.

As hypothesized, the study demonstrated that self-reported psychological distress from the pandemic was common, as well as being worried about one’s sport and about one’s own future in sport. Likewise, as hypothesized, in women, depression and anxiety were relatively common, whereas in contrast, these symptoms were rare in men. The hypothesis of gender differences was confirmed for psychological distress due to COVID-19, worry about one’s own future in sports, depression and anxiety, whereas worry about one’s sport overall was not more common in women. Also, in line with the hypothesis, depression and anxiety in the sample were significantly associated with self-reported distress due to COVID-19 and worry about one’s own situation in sports. Contrary to the hypotheses, having increased gambling or alcohol drinking was largely unrelated to perceived distress from COVID-19 (except for increased alcohol drinking being associated with worry about one’s own future in sports). The hypothesis of a gender difference in moderate-risk/problem gambling in athletes was confirmed; this was clearly more common in men than in women and thus corroborates previous findings on elevated problem gambling prevalence in male–, but not female– elite athletes [19,20].

The psychological impact of COVID-19 on athletes, theoretically a group largely affected in their every-day lives by a crisis leading to a nearly full lockdown of sports events globally, hitherto has not been reported scientifically. Thus, while comparisons to other data or similar crises is difficult, very large percentages in the present study reported some psychological influence from the current crisis; a majority reported feeling psychologically worse, although with a mild expression of this in most cases, and a majority expressed concerns about the future of their sport in this country and about their own future in the sport.

Although in different sports and different settings, a few studies in different groups of elite athletes are available for comparison. Rates of clinical depression or anxiety were high in females, but low in males in the present study. Although direct comparisons are few, in a recent report from soccer players in a number of European countries, recently reported in news media, diagnostic level of depression was reported in 22% of women and 13% of men, and the corresponding level of anxiety was reported in 18% of women and 16% of men [11]. Thus, in the present study, rates of depression and anxiety in women were comparable to this European report, although for men, figures in the present study were markedly lower. The same was seen if assessing only soccer players; for women, depression (17%) and anxiety (22%) were comparable to the European report, whereas for men, like in the overall sample, depression (five percent) and anxiety (seven percent) were low.

A recent study in elite athletes in Sweden is available for comparison [37], and in this study, anxiety measured by GAD-7 was detected in 12.6% of athletes, with higher prevalence in women (16.8%) than in men (5.6%). Depression was seen in 14.7% (20.4% of women and 5.6% of men). These rates were comparable to figures seen in the present study during the COVID-19 period. Based on this comparison, although compared to elite athletes in general rather than in the three specific sports studied here, the current findings again do not clearly demonstrate any COVID-19-related increase in anxiety or depression during this period. However, a German study in top-league female soccer players demonstrated that eight percent fulfilled the corresponding GAD-criteria, i.e., markedly lower than in the present study [41]. In addition, in a corresponding study including both men and women from top soccer leagues in Switzerland, scoring above the GAD-7 cutoff for moderate anxiety was surprisingly rare, with around one percent of male and female players reaching cutoff [42]. Previous studies have found past-month prevalence rates of up to 26% for combined depression/anxiety [43] and Nixdorf and coworkers reported a 15-percent prevalence of major depressive disorder in elite athletes [44]. Thus, figures in previous studies were inconsistent, but altogether, the prevalence rates of depression and anxiety reported in the present study, where around 13% met the past-fortnight criteria of either depression or anxiety, could be considered comparable to previous findings or possibly even towards the lower end in comparison. In summary, these findings do not demonstrate high degrees of diagnostic depression or anxiety during the COVID-19 pandemic in this sample. Meanwhile, it cannot be excluded that psychological distress may increase gradually in case of a prolonged lockdown in major sports events.

While both ice hockey and handball are sports where the COVID-19 pandemic interrupted the final phases of the season and cancelled play-off and final rounds, Sweden has a spring-to-autumn soccer session, and therefore, the pandemic has led to uncertainty in soccer players about the whole forthcoming season. In addition, the COVID-19 policies of Sweden have differed from those of most other countries, making every-life and working conditions in general significantly less impacted than in many other countries [30]. It is beyond the scope of the present study to conclude whether this may lower the rates of psychological distress in men in this population, but not in women, and it cannot be excluded that these differences are due to other factors specific to Sweden or instead due to factors related to inclusion bias in the present study. Moreover, being a professional women athlete is financially more challenging and uncertain across the three sports included in the study, especially in ice-hockey. This can potentially also explain higher levels of distress among women athletes. Further research is needed in order to outline the gender-specific effects of the COVID-19 pandemic in athletes.

In addition, it has been shown that mental health symptoms, such as depression, may vary substantially depending on whether they are assessed before or after a major competition in elite athletes; Hammond and coworkers demonstrated that rates of depression were surprisingly high prior to major performance and then were reduced to around half after the competition [45], further adding to the complexity of measuring mental health symptoms in elite athletes. The present study included three sports where seasons are diverse in Sweden, with an autumn-to-spring season for handball and ice hockey and a spring-to-autumn season in soccer. However, for obvious reasons, as the study was carried out during nearly total sports confinement due to the COVID-19 pandemic, it is very difficult to assess how the choice of period of study would affect mental health with respect to temporality in relation to competition. For ice hockey and handball, it was certain that no competition would occur for the next few months, but also with uncertainty about the season to follow, whereas for soccer, the start of the season had been postponed and the period of study can be suspected to be characterized by impatience and uncertainty even about the next few weeks. Thus, this adds to the complexity of interpreting depression and anxiety findings in relation to sports, although rates of disorders did not differ substantially across sports. Possibly, the most relevant interpretation of depression and anxiety in the current situation therefore may be the substantial gender differences and the association between clinical disorders and self-reported influence and concern related to the pandemic. Regardless of the potential association with the current crisis, the need for clinical attention to mental health in elite athletes has been highlighted during the past few years, including initiatives such as specialized treatment units for athletes with mental health problems [46], including in the present setting [47].

Problem gambling traditionally has been more common in men in the general population, but gender differences in prevalence may have become narrower during the past few years [39,40]. However, males appear to be even more over-represented with respect to problem gambling in elite athletes [19,20] and in fitness-involved young adults and adolescents [48]. In the present study, as in previous studies in athletes, problem gambling was clearly more common in men and more common in this group than likely would be the case in the general population [21,39], whereas gambling on a moderate-risk or problem level was even non-existing in women.

In recent findings, it has been demonstrated that changes in gambling behavior during the COVID-19 crisis are likely to be limited in the present setting; among general population-recruited web survey respondents, fewer respondents reported an increase in gambling during the crisis compared to those reporting a decrease, but a clear pattern was that individuals who reported having increased their overall gambling or who had transferred in their gambling behavior from the almost non-existing sports gambling to other gambling types, had considerably higher rates of gambling problems [31]. The findings here are similar; a limited number of respondents reported increased gambling behavior during the crisis and few reported an increase in specific other gambling types in response to the decrease in traditional sports betting during the confinement. Among those who did report an increase in gambling, rates of gambling problems were high. In addition, when including only those who did not report to be never-gamblers, the association with gambling problems remained, and increased gambling during the crisis also became significantly associated with increased alcohol drinking during the same period, also a finding consistent with previous general population data [31]. Thus, the present findings indicate that in a population, elite athletes, mainly in men, with a previously known risk increase in problem gambling compared to the general population [19,20], individuals who increase their gambling behavior in response to an overall crisis such as the COVID-19 pandemic may need specific preventive and supporting interventions and may be a group with a more pronounced change in life-style habits during the crisis than others. These findings further point to the importance of highlighting the connection between gambling and the world of sports and to highlight discussions around responsible gambling and gambling attitudes in male athletes.

The present findings also have research implication for various post-pandemic phases related to the ongoing development of the COVID-19 infection. The present survey was initiated due to the lockdown and very uncertain circumstances of the world of sports during the pandemic. In addition, the subsequent gradual opening of sports events and the remaining and often changing restrictions applied to public events including sports events, clearly demonstrate the need for longitudinal follow-up studies, both in samples corresponding to the one studied here and in other groups of elite athletes. For example, the findings from the present study also call for studies involving other sports, such as individual sports and Paralympic sports, also related to the postponed 2020 Olympic and Paralympic games and other major sports events.

## 5. Strengths and Limitations

The present study has limitations, mainly associated with the fact that this is an anonymous online survey, with limited possibilities for in-depth questions or full diagnostic instruments to be used and associated with the low response rate. Likewise, the present study is cross-sectional and although it lays the ground for repeated studies in the same geographical setting and with the same inclusion criteria, a longitudinal follow-up of the individuals included here would have been a strength. However, as full confidentiality was guaranteed in the present study, no personal data are available for follow-up of the same respondents as in the present study, which instead serves as a timely measure of potential reactions to an ongoing situation but calls for new and repeated studies to be carried out.

In previous studies addressing mental health in elite athletes, response rates have been variable; a survey of a population corresponding to the one studied here—members of the soccer players’ international union—reached a response rate of 29% [43]. Similar response rates were achieved in the sports assessed in the present study. In the present study, the calculation of an absolute response rate was difficult since a second pathway was for recruitment of athletes in the same target group, as the target population consists of both union members and non-members in elite clubs. In addition, it was critical to ensure full confidentiality to the respondents, given the fact that many of them may be well known to the general public; thus, a formal registration of nonrespondents was not possible. While the assessment of mental health in well-known top athletes with registered personal data may be a challenge, future studies should aim for structured follow-up of response and nonresponse rates in this population.

A low response rate may lead to a risk of inclusion bias; a survey presented to address mental health and gambling during the COVID-19 may either attract individuals who perceive a high level of impact from the pandemic, although this is difficult to demonstrate, and the opposite bias is also difficult to rule out, i.e., a possible under-reporting of psychological consequences because of a selection bias. Thus, the tendency to participate could both be enhanced by a high degree of personal involvement in debate issues related to the sports situation during COVID-19, as well as to actual feelings of personal distress. Therefore, the lack of information in the present brief web survey about personal reasons for participating is a limitation. In line with this, it could be possible to hypothesize that feelings related to the COVID-19 lockdown in sports may differ depending on one’s own status in the sports. Theoretically—and based on experience from professional practice—an athlete at the end of a long competitive season, such as in handball or ice hockey, may respond differently than an athlete waiting and eager to start the competitive season (as in soccer). In addition, athletes who struggled with injuries and other difficulties have spoken out in media about appreciating the extra time that would allow a return to sport with less stress. Again, the lack of such in-depth information in this relatively brief web survey may be seen as a limitation.

Related to the online survey design, brief instruments were used for the measures of depression and anxiety. This is also a limitation of the present study. For example, the PHQ-9 cutoff of 10 recently has been criticized for possibly being too low [49]. However, given the low amount of comparable data, the present study utilized the same instruments as in another recent report on soccer players [8], in order to facilitate comparisons. In addition, the study used established cutoff allowing comparisons with other studies in elite athletes [11,37]. The PGSI can be considered a ‘golden standard’ instrument for the measure of gambling problems in survey studies and with a high degree of comparable data sources in the population [39,40]. Likewise, the lack of exact information about age, except for the dichotomous measure of age below or above 25 years, is a limitation of the study, but was judged necessary in order to guarantee a feeling of full confidentiality to the participants, as many may be nationally or regionally well-known to sports supporters and through the media.

Strengths of the study include the fact that it assesses a timely and novel area of research, during ongoing lockdown circumstances caused by the COVID-19 pandemic. In addition, the study merits from having combined shorter and straightforward self-report items of current distress due to the pandemic, with more established and structured measures of symptoms. In addition, studying problem gambling in elite athletes can be considered a relatively novel area of research, such that studies hitherto are few, and one strength of the study was the measure of gambling problems with a well-established golden standard instrument, allowing for comparisons with other samples.

## 6. Conclusions

During the ongoing COVID-19 crisis, self-reported psychological influence from the pandemic has been common among elite soccer, ice hockey and handball athletes in Sweden—as have been concerns about one’s sport and about one’s own career related to the crisis situation. Depression and anxiety were not markedly higher than expected but were associated with COVID-19-related descriptions of worry. Fear of increased gambling during the crisis could not be clearly demonstrated, but at-risk gambling in male athletes was common and also linked to an increase in gambling during the pandemic. Psychological influence from the pandemic or other enduring crisis should be further addressed in athletes and more research is needed, including in-depth study design and studies beyond the specific sports assessed here.

## Figures and Tables

**Table 1:** Representation of sports and gender in the target population addressed with the online survey and in respondents.

Recruitment, Per Sport	Player Union Members Formally Addressed Directly (e-mail), N = 1145), n (Men, Women)	Further Recruitment Strategy	Respondents (N = 327), n (% of All Responders)	Male, Female (n) ^1^
Soccer	487 (343, 144)	Reminder through ‘What’s App’ through coordinators of separate teams (44 soccer teams)	191 (58% responders)	95, 82
Handball	140 (94, 46)	–	52 (16% of responders)	25, 25
Ice hockey	518 (499, 19)	Due to low number of female members, additional spread through coordinators of 10 female teams.	84 (26% of responders)	72, 11
All	1145 (936, 209)		327	192, 118 ^1^

^1^ gender reported by 310 individuals (missing for 17 cases).

**Table 2:** Sample characteristics.

Sample Characteristics	N = 327,% (n)	Missing Data (n)
Sports		0
- Soccer - Ice hockey - Handball	58 (191) 26 (184) 16 (52)	
Gender		17
- Female - Male	38 (118) 62 (192)	
Age		17
- Below 25 years - 25 years or above	42 (131) 58 (179)	
Mental state during COVID-19		31
- Better or unaffected - Slightly worse - Much worse	48 (143) 46 (135) 6 (18)	
Thoughts on the future of one’s sport		32
- Not worried - Slightly worried - Very worried	34 (100) 60 (178) 6 (17)	
Thoughts on one’s own future in the sport		32
- Not worried or neither - Slightly worried - Very worried	48 (143) 42 (123) 9 (27)	
Alcohol consumption during COVID-19		50
- Does not drink (neither now, nor before) - Drinks less - Unchanged - Drinks more	13 (37) 13 (36) 57 (159) 16 (45)	
Gambling during COVID-19		50
- Does not gamble (neither now, nor before) - Gambles less - Unchanged - Gambles more	48 (134) 12 (32) 33 (91) 7 (20)	
Depression		48
- Yes (PHQ-9, score > 9) - No	9 (25) 91 (254)	
Anxiety		41
- Yes (GAD-7, score > 9) - No	10 (30) 90 (256)	
Level of gambling problems		53
- No risk - Low risk - Moderate risk - Problem gambling	86 (236) 7 (20) 5 (15) 1 (3)	

**Table 3:** Fulfillment of at least moderate depression symptoms (patient health questionnaire-9 score of 10 or above), depending on other variables. Chi-squared analyses for group comparisons and logistic regression analyses for the analysis of each potential correlate when controlling for gender.

Group Characteristics	Depression	No Depression	*p* Value	*p* Value Controlling for Gender ^3^
Sports			0.20	0.79
- Soccer - Ice hockey - Handball	10% (16) 4% (3) 13% ^4^ (6)	90% (141) 96% (71) 88% ^4^ (42)		
Gender			<0.001	–
- Female - Male	19% (19) 3% (6)	81% (83) 97% (171)		
Age			0.77	0.72
- Below 25 years - 25 or above	10% (11) 9% (14)	90% (104) 91% (150)		
Mental state during COVID-19			<0.001 ^1^	<0.001
- Better or unaffected - Slightly worse - Much worse	2% (2) 11% (14) 56% (9)	98% (131) 89% (116) 44% (7)		
Thoughts on the future of one’s sport			0.26 ^1^	0.50
- Not worried - Slightly worried - Very worried	6% (6) 10% (17) 12% (2)	94% (90) 90% (149) 88% (15)		
Thoughts on one’s own future in the sport			<0.001 ^1^	<0.01
- Not worried - Slightly worried - Very worried	3% (4) 12% (14) 27% (7)	97% (133) 88% (102) 73% (19)		
Drinks more alcohol during COVID-19			0.22	0.29
- Yes - No	13% (6) 8% (18)	87% (39) 92% (214)		
Gambles more during COVID-19			0.69 ^2^	0.48
- Yes - No	10% (2) 9% (22)	90% (18) 91% (235)		

^1^ chi-squared, linear-by-linear. ^2^ Fisher’s exact test. ^3^ logistic regression controlling for gender. ^4^ Rounded off to whole percentages; thus, sum does not add up to 100%.

**Table 4:** Fulfillment of at least moderate anxiety symptoms (GAD-7 score of 10 or above), depending on other variables. Chi-squared analyses for group comparisons and logistic regression analyses for the analysis of each potential correlate when controlling for gender.

Group Characteristics	Anxiety	NO Anxiety	*p* Value	*p* Value Controlling for Gender ^3^
Sports				
- Soccer - Ice hockey - Handball	14% (22) 4% (3) 10% (5)	86% (139) 96% (74) 90% (43)	0.07	0.17
Gender				
- Female - Male	20% (21) 5% (9)	80% (83) 95% (173)	<0.001	-
Age				
- Below 25 years - 25 or above	10% (12) 11% (18)	90% (107) 89% (149)	0.85	0.43
Mental state during COVID-19				
- Better or unaffected - Slightly worse - Much worse	1% (1) 15% (20) 56% (9)	99% (137) 85% (112) 44% (7)	<0.001 ^1^	<0.001
Thoughts on the future of one’s sport				
- Not worried - Slightly worried - Very worried	8% (8) 12% (20) 12% (2)	92% (90) 88% (151) 88% (15)	0.40 ^1^	0.65
Thoughts on one’s own future in the sport				
- Not worried - Slightly worried - Very worried	5% (7) 10% (12) 42% (11)	95% (133) 90% (108) 58% (15)	<0.001 ^1^	<0.001
Drinks more alcohol during COVID-19				
- Yes - No	16% (7) 9% (22)	84% (38) 91% (210)	0.22	0.28
Gambles more during COVID-19				
- Yes - No	15% (3) 10% (26)	85% (17) 90% (231)	0.45 ^2^	0.24

^1^ chi-squared, linear-by-linear. ^2^ Fisher’s exact test. ^3^ logistic regression controlling for gender.

**Table 5:** Self-reported increase in gambling during the COVID-19 pandemic, depending on other variables. Chi-squared analyses for group comparisons.

Group Characteristics	Increased Gambling	No Increased Gambling	*p* Value
Sports			
- Soccer - Ice hockey - Handball	6% (9) 7% (5) 13% (6)	94% (146) 93% (69) 87% (42)	0.29
Gender			
- Female - Male	4% (4) 9% (16)	96% (96) 91% (161)	0.15 ^2^
Age			
- Below 25 years - 25 or above	6% (7) 8% (13)	94% (107) 92% (150)	0.56
Mental state during COVID-19			
- Better or unaffected - Slightly worse - Much worse	6% (8) 9% (12) 0% (0)	94% (125) 91% (116) 100% (16)	0.86 ^1^
Thoughts on the future of one’s sport			
- Not worried - Slightly worried - Very worried	7% (7) 7% (12) 6% (1)	93% (88) 93% (153) 94% (16)	0.88 ^1^
Thoughts on one’s own future in the sport			
- Not worried - Slightly worried - Very worried	9% (12) 5% (6) 8% (2)	91% (124) 95% (109) 92% (24)	0.47 ^1^
Drinks more alcohol during COVID-19			
- Yes - No	13% (6) 6% (14)	87% (39) 94% (218)	0.08
Anxiety above cutoff			
- Yes - No	10% (3) 7% (17)	90% (26) 93% (231)	0.45 ^2^
Depression above cutoff			
- Yes - No	8% (2) 7% (18)	92% (22) 93% (235)	0.69 ^2^
Gambling severity level			
- No risk - Low risk - Moderate risk - Problem gambling	5% (11) 20% (4) 33% (5) 0% (0)	95% (225) 80% (16) 67% (10) 100% (3)	<0.001 ^1^

^1^ chi-squared, linear-by-linear. ^2^ Fisher’s exact test.

**Table 6:** Self-reported increase in alcohol consumption during the COVID-19 pandemic, depending on other variables. Chi-squared analyses for group comparisons.

Group Characteristics	Increased Alcohol Drinking	No Increased Alcohol Drinking	*p* Value
Sports			
- Soccer - Ice hockey - Handball	15% (23) 12% (9) 27% (13)	85% (132) 88% (65) 73% (35)	0.07
Gender			
- Female - Male	18% (18) 15% (27)	82% (82) 85% (150)	0.55
Age			
- Below 25 years - 25 or above	18% (21) 15% (24)	82% (93) 85% (139)	0.41
Mental state during COVID-19			
- Better or unaffected - Slightly worse - Much worse	14% (18) 20% (26) 6% (1)	86% (115) 80% (102) 94% (15)	0.59 ^1^
Thoughts on the future of one’s sport			
- Not worried - Slightly worried - Very worried	8% (8) 20% (33) 24% (4)	92% (87) 80% (132) 76% (13)	0.01 ^1^
Thoughts on one’s own future in the sport			
- Not worried - Slightly worried - Very worried	13% (17) 20% (23) 19% (5)	87% (119) 80% (92) 81% (21)	0.14 ^1^
Anxiety above cutoff			
- Yes - No	24% (7) 15% (38)	76% (22) 85% (210)	0.22
Depression above cutoff			
- Yes - No	25% (6) 15% (39)	75% (18) 85% (214)	0.22
Gambles more during COVID-19			
- Yes - No	30% (6) 15% (39)	70% (14) 85% (218)	0.08

^1^ chi-squared, linear-by-linear.
